# Thyroid disorders and pregnancy loss: a bidirectional mendelian randomization exploration

**DOI:** 10.1186/s12884-025-08146-2

**Published:** 2025-10-14

**Authors:** Panyu Chen, Weie Zhao, Yujie Li, Lei Jia, Cong Fang, Manchao Li

**Affiliations:** 1 Department of Reproductive Medicine Center, the Sixth Affiliated Hospital of Sun Yat-sen University, Guangdong Engineering Technology Research Center of Fertility Preservation, Guangzhou, Guangdong China; 2 Guangdong Engineering Technology Research Center of Fertility Preservation, Guangzhou, Guangdong China; 3 Biomedical Innovation Center, The Sixth Affiliated Hospital, Sun Yat-sen University, Guangzhou, Guangdong 510000 China

**Keywords:** Hyperthyroidism, Hypothyroidism, Spontaneous abortion, Recurrent spontaneous abortion, Mendelian randomization

## Abstract

**Objective:**

Thyroid hormones are essential for normal pregnancy and fetal development. The relationship between thyroid disease and the risk of spontaneous abortion (SA) and recurrent spontaneous abortion (RSA) has been reported, but causality is unclear. The purpose of this study was to investigate whether there is a causal association between thyroid disorders and SA/RSA.

**Methods:**

In this bidirectional mendelian randomization (MR) analysis, the causal effects of genetically predicted hyperthyroidism, hypothyroidism on adverse pregnancy outcome including SA and RSA were explored. Statistics on thyroid disorders-associated genetic variants were obtained from the Sakaue’s genome-wide association studies (GWAS). The SA and RSA GWAS from the Laisk’s study and FinnGen cohort were used as outcome data for discovery and replication analyses, respectively. Results were pooled by meta-analysis. A reverse MR analysis was also conducted by treating SA/RSA as the exposure to further substantiate our results.

**Results:**

Genetically predicted hypothyroidism showed a modest positive association with SA (OR: 1.024, 95% CI: 1.005–1.043, *P* = 0.014), though the effect size was small. No significant associations of hypothyroidism with RSA, hyperthyroidism with SA or RSA were found (all *P* > 0.05). Reverse MR and sensitivity analyses further confirmed the robustness of the results.

**Conclusions:**

This study suggests a potential causal role of hypothyroidism in SA, albeit with a limited effect magnitude. There were no significant causal relationships of hypothyroidism with RSA, hyperthyroidism with SA or RSA and SA/RSA with thyroid disorders. Further studies should validate these findings and elucidate the pathophysiological mechanisms behind them.

**Supplementary Information:**

The online version contains supplementary material available at 10.1186/s12884-025-08146-2.

## Introduction

The adaptive changes in thyroid function during pregnancy primarily manifest as thyroid gland enlargement. This compensatory morphological alteration directly supports the increased demand for thyroid hormone synthesis, with its fundamental physiological significance lying in maintaining serum free thyroxine (fT4) levels within normal ranges. This regulatory process involves coordinated participation of multiple mechanisms: Pregnancy-associated hormonal changes not only induce a remarkable 50% elevation in thyroxine-binding globulin (TBG) levels [1], but also accelerate thyroid hormone metabolism through upregulation of placenta-specific deiodinase DIO3 activity, accompanied by a significant 30–50% enhancement in renal iodine excretion efficiency compared to baseline levels. Furthermore, the direct stimulatory effect of human chorionic gonadotropin (hCG) on the thyroid gland warrants particular attention.

Pregnancy essentially serves as a biological stress test for thyroid function. In women with no preexisting thyroid pathology (defined as euthyroid status with negative thyroid antibodies), the thyroid gland demonstrates remarkable compensatory capacity. However, those with diminished thyroid reserve—particularly women with Hashimoto’s thyroiditis (an autoimmune disorder defined by TPO antibody positivity and lymphocytic infiltration of the thyroid gland)—often cannot meet the increased hormonal demands of gestation, potentially developing subclinical or overt hypothyroidism. Similarly, conditions like Graves’ disease and thyroid nodules can disrupt normal thyroid hormone synthesis and secretion, potentially resulting in hyperthyroidism during pregnancy [2].

Accumulating clinical evidence demonstrates that maternal thyroid dysfunction during pregnancy is significantly associated with adverse outcomes affecting both maternal and fetal health. Multiple observational studies have consistently shown that overt hypothyroidism and hyperthyroidism are linked to an increased risk of spontaneous abortion (SA), gestational hypertension, preeclampsia, and preterm delivery [1]. A study reporting the relationship between untreated hypothyroidism and SA showed that women with untreated hypothyroidism had a higher risk of SA compared with controls with normal thyroid function [2]. Another study of 240 patients with subclinical hypothyroidism and 10,518 control patients showed no difference in SA rates [3]. Studies on recurrent spontaneous abortion (RSA) and thyroid disorders have been scarce. Only one study has concluded that thyroid disorders do not lead to an increased risk of RSA [4]. Current evidence remains inconclusive regarding the thyroid disorder-SA/RSA relationship, as existing observational studies are inherently limited by potential confounding factors and cannot establish causal inference. To address these methodological constraints, we conducted a bidirectional two-sample Mendelian randomization (MR) analysis to: (1) investigate the potential causal effects of thyroid dysfunction on both SA and RSA, and (2) perform reverse MR to examine whether SA/RSA might reciprocally influence thyroid disorder risk.

## Materials and methods

### Study design

MR is a method that uses measured genetic variation to study the causal effect of an exposure on an outcome. It has three core instrumental variable assumptions (1). The genetic variant(s) being used as an instrumental variable for the exposure needs to be highly correlated with the exposure. This is the “relevance” assumption (2). There are no confounders of the genetic variant(s) and the outcome of interest. This is the “independence” or “exchangeability” assumption (3). Genetic variation(s) can only act through the one and only pathway of exposure to the outcome. This is the “exclusion restriction” or “no horizontal pleiotropy” assumption [5].

**Fig. 1:**
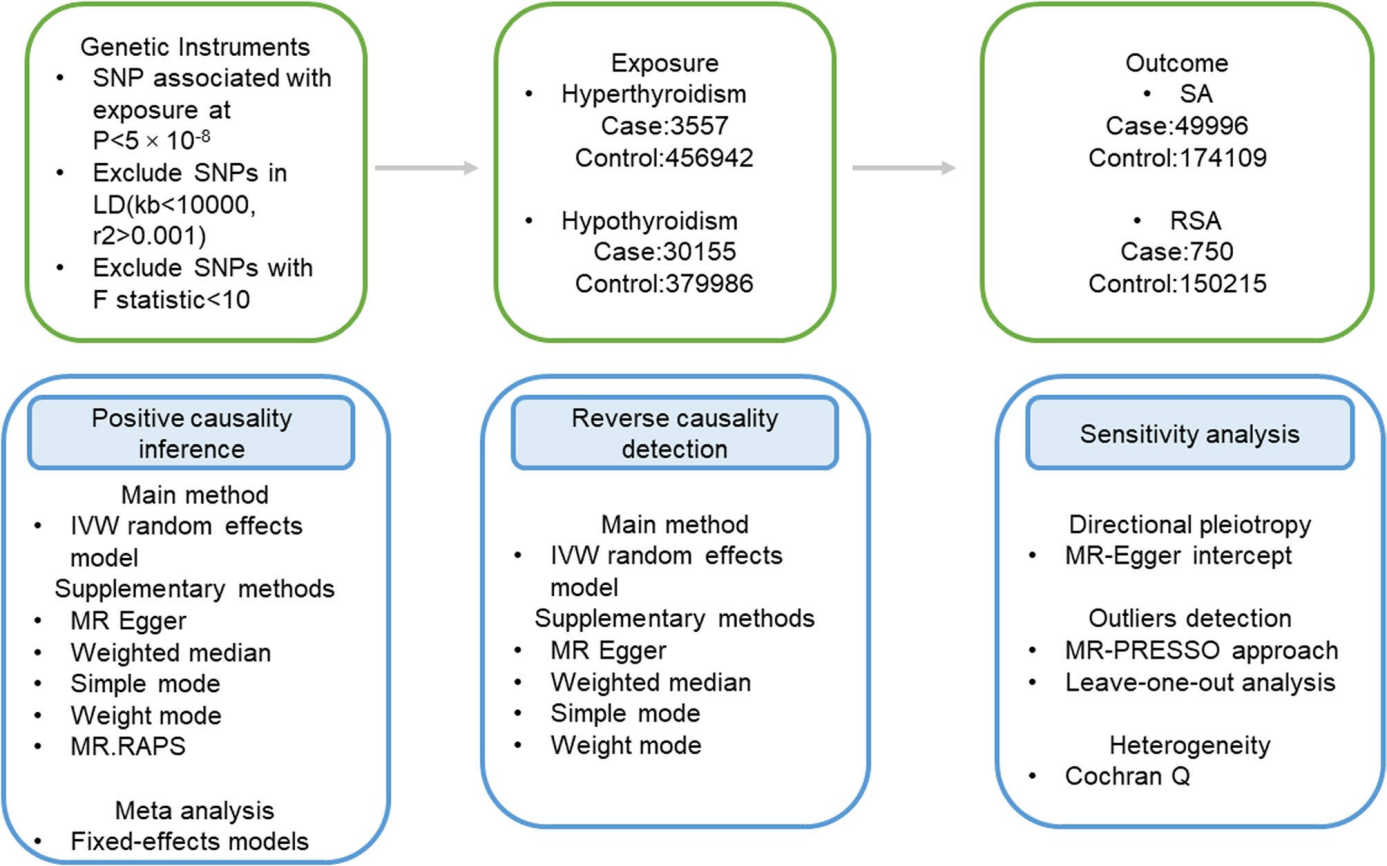
Flow chart of the study design. SA, spontaneous abortion; RSA, recurrent spontaneous abortion

Our MR study evaluates the causal association of thyroid disorders including hypothyroidism/hyperthyroidism and pregnancy outcomes including SA and RSA, as illustrated in Fig. 1.

### Study population

We obtained summary-level genetic data for thyroid disorders from the European-ancestry GWAS by Sakaue et al., comprising 30,155 hypothyroidism cases versus 379,986 controls and 3,557 hyperthyroidism cases versus 456,942 controls [6]. The thyroid disorders dataset was derived from a meta-analysis of UK Biobank (UKB) and FinnGen data, comprising 3,557 cases and 456,942 controls of European ancestry. The UKB cohort recruited approximately 500,000 participants across the United Kingdom, with a mean enrollment age of 56.8 years (53.8% female). FinnGen represents a public-private partnership integrating Finnish biobank genetic data with national health registries, featuring participants with a mean age of 51.8 years at DNA sampling (56.3% female). Pregnancy outcome data were sourced from the large-scale European-ancestry GWAS by Laisk et al., including 49,996 SA cases versus 174,109 controls and 750 recurrent spontaneous abortion RSA cases versus 150,215 controls [7]. SA was classified as 1–2 pregnancy losses, whereas RSA was defined as ≥ 3 consecutive self-reported miscarriages. We employed MR-Lap to quantify sample overlap between thyroid dysfunction and SA/RSA. Based on the computed intercept values (all < 0.01), we concluded there was no substantial sample overlap between the GWAS datasets for thyroid dysfunction and abortion outcomes [8]. For replication analyses, we utilized additional summary statistics from the FinnGen Release 3 dataset.

### Instrumental variables (IVs) selection

Single nucleotide polymorphisms (SNPs) correlated with exposures with genome-wide significance (*P* < 5 × 10 − 8) were screened out firstly. Then, they were filtered with LD-based clumping to screen independent ones within a threshold of, 10,000 kb and r2 < 0.001. When F-statistics is < 10, This instrumental variable will be excluded as a weak instrumental variable.

### Two-sample MR

To examine the potential causal effects of thyroid disorders on SA and RSA, we performed a two-sample MR analysis using genetic instruments derived from the aforementioned GWAS summary statistics. The primary causal estimates were obtained using a random-effects inverse-variance weighted (IVW) approach, supplemented by additional MR methods, including MR-Egger, simple mode, weighted median, and weighted mode, to enhance robustness. Given its ability to account for systematic and eccentric pleiotropy, the robust adjusted profile score (RAPS) method was also applied to strengthen causal inference, particularly in scenarios involving many weak instruments. To further assess the validity of our findings, we conducted sensitivity analyses using the MR-Egger intercept test [9] and the MR Pleiotropy RESidual Sum and Outlier (MR-PRESSO) global test to evaluate potential horizontal pleiotropy [10, 11]. Heterogeneity among genetic instruments for each exposure was quantified using Cochran’s Q statistic. Finally, we combined results from discovery and replication analyses through meta-analysis to derive pooled effect estimates, thereby enhancing the reliability of our conclusions [12]. All statistical analyses were carried out using the ‘DrugtargetMR’ [13], ‘TwoSampleMR’, ‘MR-PRESSO’ and ‘mr.raps’ packages in R version 4.2.2, and all P-values were two-sided.

### Bi-directional MR

We conducted Bi-directional MR to test for reverse causality. The aim of this analysis is to examine if genetic susceptibility to SA or RSA has an impact on thyroid disorders.

### Institutional review board (IRB) approval (or waiver) statement

No individual-level data were involved in this study. Hence, ethical approval was not required.

## Results

9 to 12 and 63 to 68 SNPs were considered as potential instruments for the hyperthyroidism or hypothyroidism and SA/RSA, respectively. F-statistics of these SNPs were all greater than 10 to ensure the exclusions for bias from the weak instrumental variables. (Supplementary Data Sheet 1–4).

**Fig. 2:**
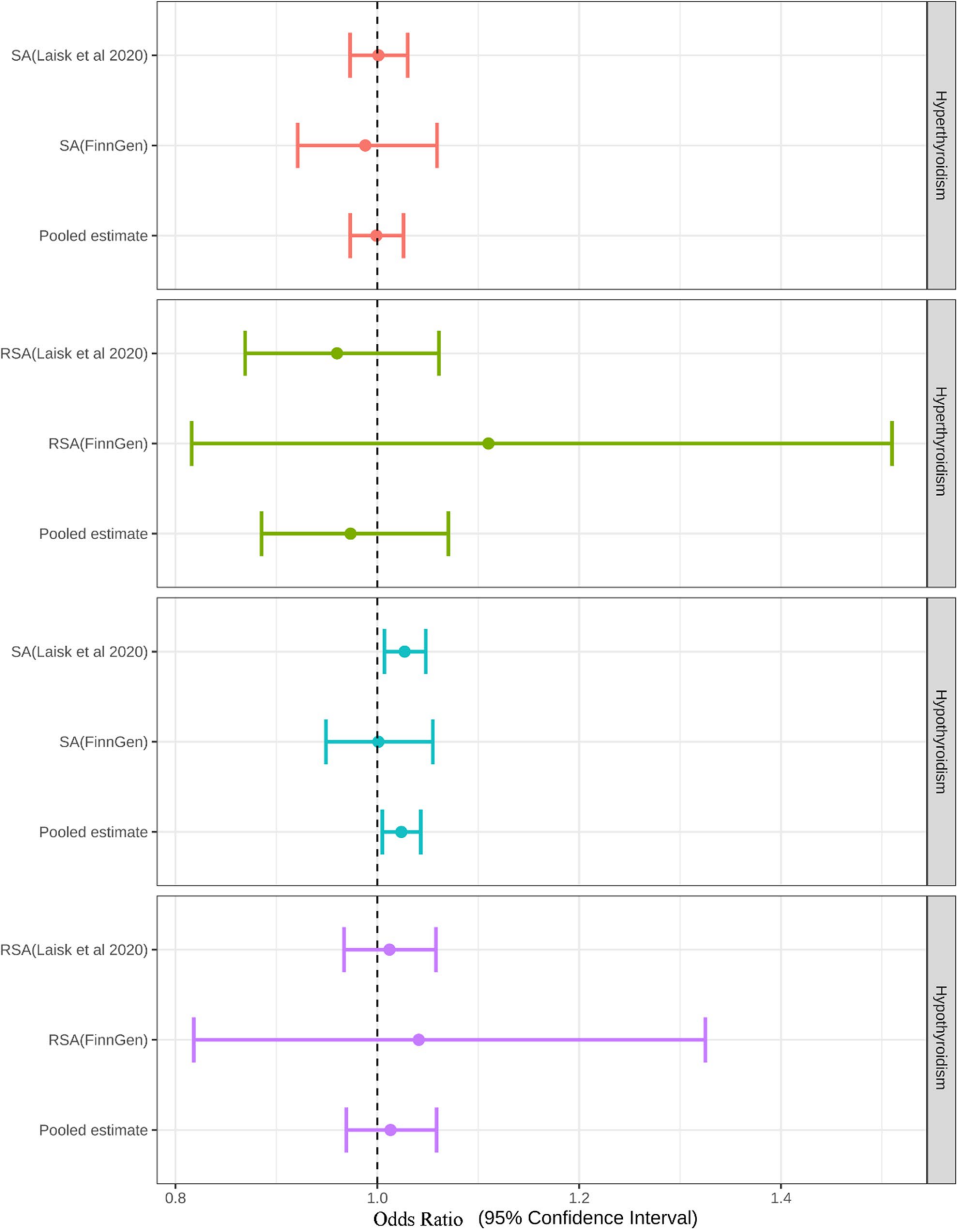
Forest plots showing results from MR of Hyperthyroidism and hypothyroidism on SA/RSA; MR, mendelian randomization, SA, spontaneous abortion; RSA, recurrent spontaneous abortion

Genetically predicted hypothyroidism was positively associated with SA in the Sakaue’s study (OR:1.027,95%CI (1.007–1.048); *P* = 0.007). The final estimate of the meta-analysis yielded similar positive results (OR:1.024,95%CI (1.005–1.043); *P* = 0.0138). No notable associations could be found between hypothyroidism with RSA, hyperthyroidism with SA and RSA (Results shown in Table 1; Fig. 2).

**Table 1:** OR table of bidirectional Mendelian randomization

Group	Exposure	Exposure ID	Outcome	Outcome ID	SNPs	Method	OR	95%CI lower	95%CI upper	*P*-value
Discovery	Hyperthyroidism	ebi-a-GCST90018860	SA	ebi-a-GCST011888	12	IVW	1.001	0.973	1.030	0.919
Replication	Hyperthyroidism	ebi-a-GCST90018860	SA	finn-b-O15_ABORT_SPONTAN	11	IVW	0.988	0.921	1.059	0.730
META	Hyperthyroidism	/	SA	/	/	/	0.999	0.973	1.026	0.950
Discovery	Hypothyroidism	ebi-a-GCST90018862	SA	ebi-a-GCST011888	68	IVW	1.027	1.007	1.048	0.008
Replication	Hypothyroidism	ebi-a-GCST90018862	SA	finn-b-O15_ABORT_SPONTAN	68	IVW	1.001	0.949	1.055	0.974
META	Hypothyroidism	/	SA	/	/	/	1.024	1.005	1.043	0.0138
Discovery	Hyperthyroidism	ebi-a-GCST90018860	RSA	/	9	IVW	1.006	0.947	1.069	0.840
Replication	Hyperthyroidism	ebi-a-GCST90018860	RSA	finn-b-N14_HABITABORT	11	IVW	1.110	0.816	1.510	0.505
META	Hyperthyroidism	/	RSA	/	/	/	1.010	0.951	1.072	0.750
Discovery	Hypothyroidism	ebi-a-GCST90018862	RSA	/	63	IVW	0.994	0.954	1.037	0.805
Replication	Hypothyroidism	ebi-a-GCST90018862	RSA	finn-b-N14_HABITABORT	68	IVW	1.041	0.818	1.325	0.745
META	Hypothyroidism	/	RSA	/	/	/	0.996	0.956	1.038	0.860

*OR* Odds Ratio, *CI* Confidence Interval, *SNPs* Single Nucleotide Polymorphisms, *SA* Spontaneous abortion, *RSA* Recurrent spontaneous abortion, *IVW* Inverse variance weighted

As shown in Supplementary Data Sheet 5, we did not detect significant causal effects of the risk of RSA on thyroid disorders. Reverse MR of SA could not be performed due to no SNP left after clumping even we set the r^2^ at 0.1 and clump window at 1000 kb.

**Fig. 3:**
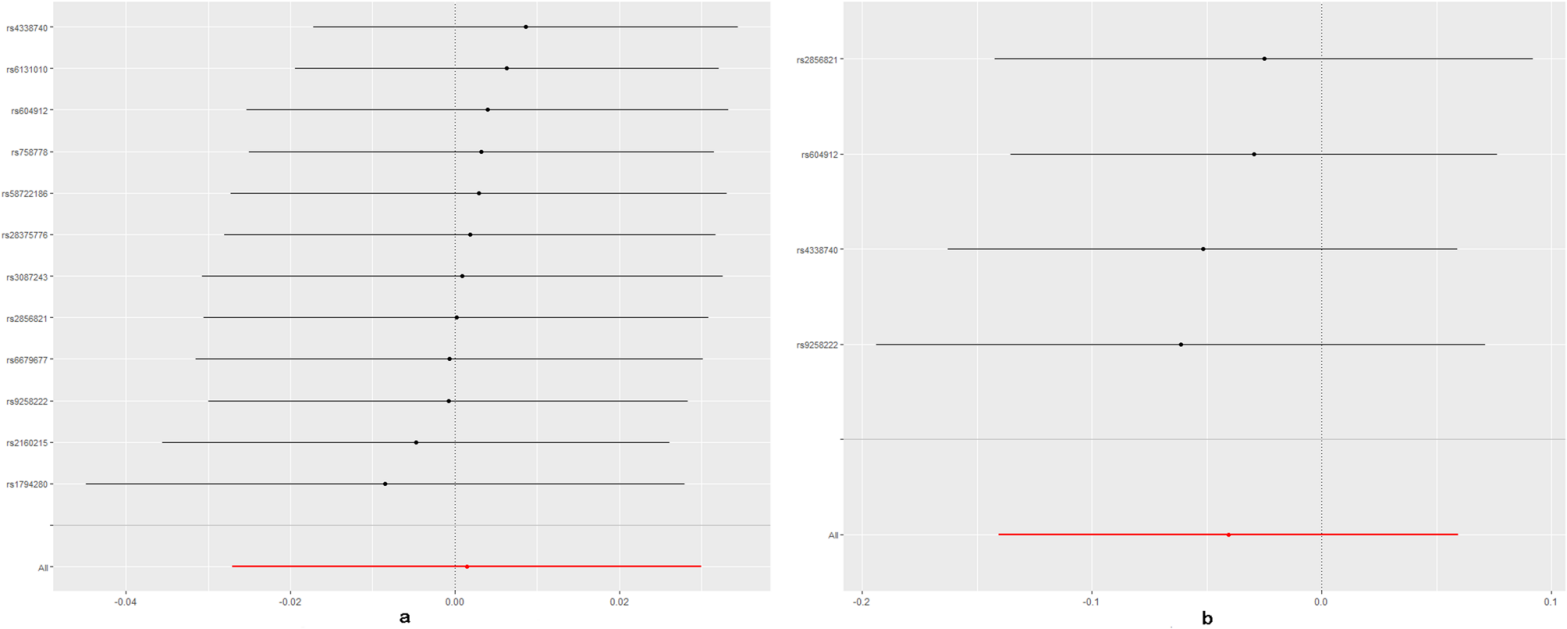
Leave-one-out plots of Hyperthyroidism on SA/RSA. SA, spontaneous abortion; RSA, recurrent spontaneous abortion.

**Fig. 4:**
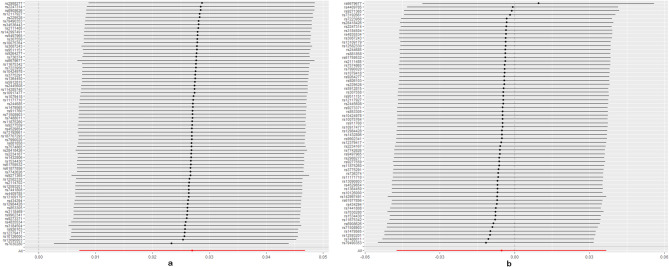
Leave-one-out plots of Hypothyroidism on SA/RSA. SA, spontaneous abortion; RSA, recurrent spontaneous abortion

**Fig. 5:**
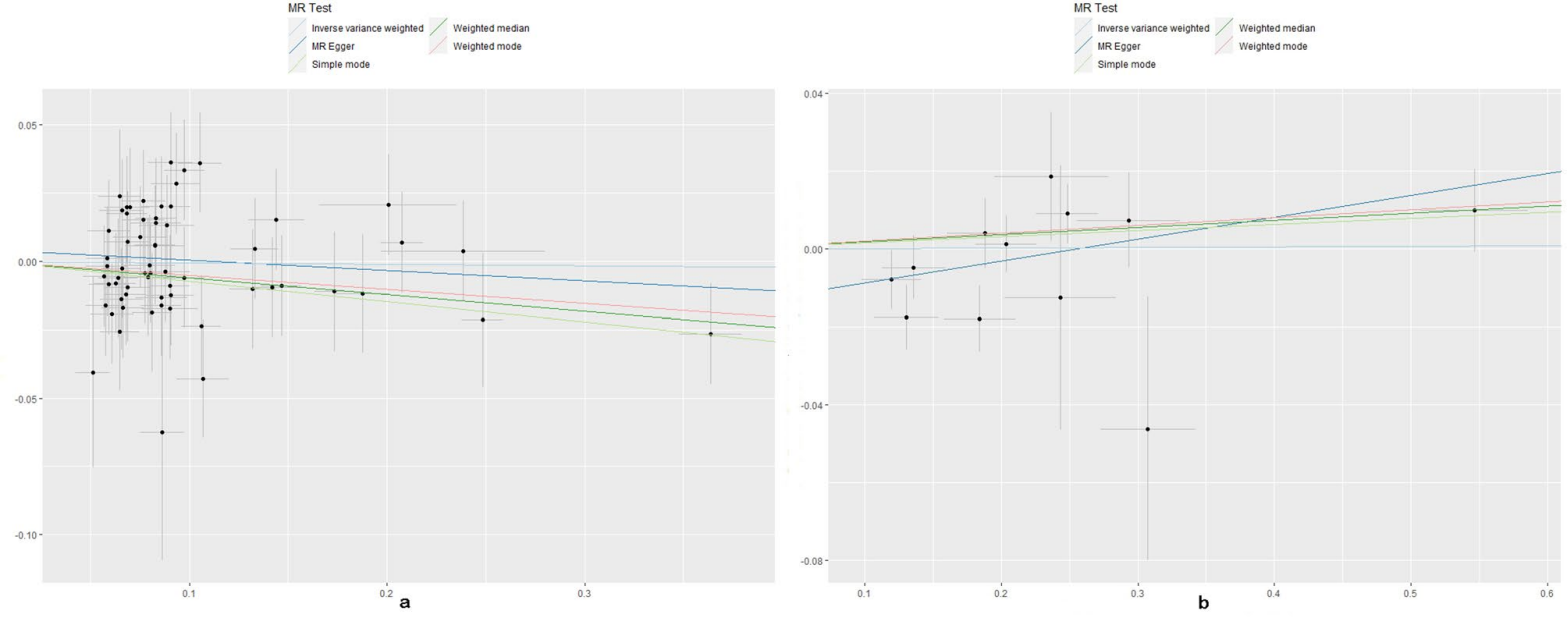
Scatter plots of Hyperthyroidism on SA/RSA. SA, spontaneous abortion; RSA, recurrent spontaneous abortion

**Fig. 6:**
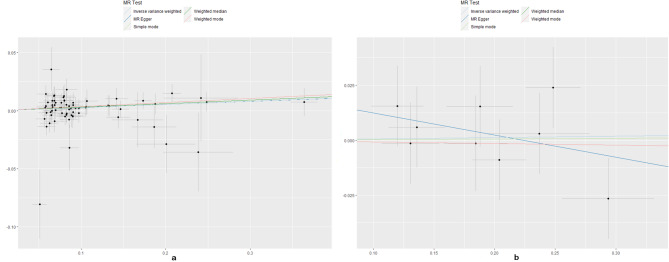
Scatter plots of Hypothyroidism on SA/RSA. SA, spontaneous abortion; RSA, recurrent spontaneous abortion

No evidence for directional pleiotropy was found when MR-Egger regression was performed (all *P* > 0.05). MR-PRESSO analyses showed no outlier SNPs in our study (Supplementary Data Sheet 6). There was no heterogeneity confirmed by both IVW and MR Egger analyses in Cochrane’s Q test with P greater than 0.05 in discovery analysis (Supplementary Data Sheet 7). Heterogeneity was found in the replication analysis of hypothyroidism/hyperthyroidism and SA using Finngen data (Supplementary Data Sheet 7). The causal estimates were unlikely to be affected by certain SNPs indicated by the leave-one-out plots (Figs. 3 and 4). In addition, the SNP effects produced by each MR method individually and jointly are demonstrated in scatter plots (Figs. 5 and 6). The results of RAPS also showed causal relationship of hypothyroidism on SA (*P* = 0.00488). No horizontal pleiotropy was detected since the estimated overdispersion parameters calculated by RAPS were all very small (Supplementary Data Sheet 8).

## Discussion

Our MR analysis identified a modest genetic association between genetically predicted hypothyroidism and SA, while revealing no significant associations for hypothyroidism with RSA or for hyperthyroidism with either outcome. The bidirectional MR analyses additionally showed no evidence of abortion outcomes influencing subsequent thyroid dysfunction risk.

Our MR findings suggest a potential causal relationship between genetically predicted hypothyroidism and spontaneous abortion. During gestation, maternal thyroid hormones play a critical role in fetal development, particularly before 18–20 weeks of gestation when fetal thyroid function becomes mature. The first-trimester placenta and developing fetus rely entirely on maternal thyroid hormone supply, which is progressively depleted through multiple physiological mechanisms: elevated thyroxine-binding globulin concentrations, enhanced renal iodine clearance, and accelerated hormone degradation by placental type 3 deiodinase [14]. These processes collectively necessitate increased maternal thyroid hormone production, supported in part by the thyrotropic effects of human chorionic gonadotropin (hCG) - a weak thyroid-stimulating hormone receptor agonist [15]. This pregnancy-induced physiological stress may unmask or exacerbate pre-existing subclinical thyroid dysfunction, potentially explaining the observed association between thyroid disorders and compromised pregnancy outcomes.

Accumulating evidence over the past twenty years has established a significant association between maternal thyroid dysfunction and adverse pregnancy outcomes, including compromised fetal development. Clinical hypothyroidism, characterized by elevated thyroid-stimulating hormone (TSH) and reduced fT4 levels, affects approximately 0.2%−0.6% of pregnant women [14, 16]. Multiple observational studies have consistently demonstrated this association, with untreated hypothyroid patients exhibiting significantly higher miscarriage rates compared to euthyroid controls [2]. These findings are further supported by prospective cohort data from Liu et al. [17] and retrospective analyses by Wang et al. [18], both reporting elevated miscarriage risk in hypothyroid pregnancies - results that align with our current investigation. The underlying pathophysiology may involve TSH receptor antibodies competitively binding to luteinizing hormone (LH) receptors in granulosa luteal cells, thereby impairing progesterone synthesis and compromising corpus luteum function during early pregnancy, ultimately leading to pregnancy loss [19].

Our MR analysis found no significant causal link between genetically predicted hypothyroidism and RSA. Current evidence regarding the association between overt hypothyroidism and RSA remains scarce, as existing studies have primarily focused on investigating subclinical hypothyroidism in relation to RSA. Existing evidence from studies of subclinical hypothyroidism presents conflicting results: while Bernardi et al. [20] and van Dijk et al. [21] reported comparable live birth rates between euthyroid women and those with subclinical hypothyroidism, and Uchida et al. [22] found no difference in RSA rates despite higher antinuclear antibody positivity in subclinical hypothyroid patients, Triggianese et al. [23] observed a significantly higher prevalence of subclinical hypothyroidism in RSA populations. These observational findings, however, cannot establish causality, highlighting the need for further research to clarify the precise relationship between thyroid dysfunction and RSA. The observed differential causal relationships between genetically predicted hypothyroidism and SA versus RSA warrant explanation from both biological and phenotypic heterogeneity perspectives. From a biological standpoint, hypothyroidism primarily disrupts early embryo implantation and placentation through maternal thyroid hormone deficiency - a mechanism that likely predominates in sporadic cases. In contrast, RSA typically involves a more complex etiological spectrum encompassing embryonic chromosomal abnormalities, uterine anatomical defects, and autoimmune disorders, where the modest effect size of thyroid dysfunction may be masked by these stronger pathogenic factors. Phenotypically, our stringent RSA definition (≥ 3 consecutive losses) may have selected for extreme phenotypes with higher genetic load or distinct pathological mechanisms, fundamentally differing from spontaneous abortion in etiological composition. Additionally, statistical power limitations cannot be overlooked - the smaller RSA sample size (*n* = 750 cases vs. 49,996 SA cases) may have precluded detection of subtle effects (e.g., OR = 1.02 for hypothyroidism). Future large-scale prospective studies incorporating longitudinal thyroid function monitoring and embryonic genetic analysis are needed to validate these mechanistic differences.

Hyperthyroidism complicates approximately 1–3% of pregnancies [24], yet its association with SA remains poorly understood, with only two studies published in 2011 and 2014 addressing this relationship. While Andersen et al. reported increased SA frequency among hyperthyroid women [25], Nangia et al. failed to replicate this association [26]. Notably, no studies have specifically examined hyperthyroidism in RSA populations. These inconsistent findings and paucity of research motivated our MR investigation to establish causal - rather than merely associative - relationships between hyperthyroidism and SA/RSA. Contrary to some observational reports, our MR study yielded negative findings that challenge previous conclusions, offering novel perspectives on this controversial topic. Importantly, while observational studies may be confounded by factors such as maternal age, body mass index (BMI), paternal contributions, or unmeasured variables, our MR approach substantially mitigates such biases, providing more reliable causal inference.

The current study has several important limitations. First, our analyses were restricted to individuals of European ancestry, limiting the generalizability of our findings to other populations. Second, the lack of sex-stratified summary statistics for hypothyroidism and hyperthyroidism genetic instruments may introduce bias and affect result precision. Additionally, while we employed multiple MR methods to enhance robustness, inherent limitations of MR—including potential pleiotropy and heterogeneity—remain unavoidable. Although two-sample MR offers advantages over randomized controlled trials (RCTs) for causal inference in observational settings, it cannot fully replace RCTs in establishing definitive causality.

In conclusion, our MR study identifies a modest genetic association between hypothyroidism and spontaneous abortion (OR: 1.024, 95% CI: 1.005–1.043), while showing no significant associations for hyperthyroidism with SA or any thyroid dysfunction with RSA. The consistency across sensitivity analyses supports the robustness of these observations, though the small effect size warrants cautious interpretation. These findings suggest a potential - yet mechanistically unclear - role of maternal hypothyroidism in sporadic pregnancy loss, distinct from recurrent miscarriage contexts. Future studies should incorporate longitudinal thyroid monitoring, detailed embryonic genetic assessments, and larger cohorts to clarify whether this association reflects biological causation or residual confounding.

## Supplementary Information

Supplementary Material 1.

Supplementary Material 2.

Supplementary Material 3.

Supplementary Material 4.

Supplementary Material 5.

Supplementary Material 6.

Supplementary Material 7.

Supplementary Material 8.

## Data Availability

GWAS summary statistics analyzed in this study are publicly available and can be found at: hypothyroidism/hyperthyroidism: https://pubmed.ncbi.nlm.nih.gov/34594039/; SA/RSA: https://pubmed.ncbi.nlm.nih.gov/33239672/; miscarriage data in replication analysis:https://gwas.mrcieu.ac.uk/datasets/finn-b-O15_ABORT_SPONTAN/

## Abbreviations

SA: Spontaneous abortion
RSA: Recurrent spontaneous abortion
MR: Mendelian randomization
GWAS: Genome-wide association studies
fT4: Free thyroxine
TBG: Thyroxine-binding globulin
hCG: Human chorionic gonadotropin
UKB: UK Biobank
IVW: Inverse-variance weighted
RAPS: Robust adjusted profile score
MR-PRESSO: MR Pleiotropy RESidual Sum and Outlier
TSH: Thyroid-stimulating hormone
LH: Luteinizing hormone
BMI: Body mass index
RCTs: Randomized controlled trials
